# Induction of granulation tissue for the secretion of growth factors and the promotion of bone defect repair

**DOI:** 10.1186/s13018-015-0287-4

**Published:** 2015-09-17

**Authors:** Xiaohua Wang, Fuda Wei, Fei Luo, Ke Huang, Zhao Xie

**Affiliations:** National & Regional United Engineering Laboratory of Tissue Engineering, Department of Orthopaedics, Southwest Hospital, Third Military Medical University, Chongqing, 400038 The People’s Republic of China

**Keywords:** Induced membrane, Periosteum, Growth factor

## Abstract

**Background:**

The use of the Masquelet technique in the repair of large bone defects has gained increased acceptance in recent years. The core of this technique is the induction of granulation tissue membrane formation and the implantation of an autologous cancellous bone to reconstruct bone defects in the membrane. In this study, we purpose to explore the structure of induced membrane and the content of growth factors as well to compare between the structure and the effects on osteogenesis of induced membranes and the periosteum in animal models.

**Methods:**

Bilateral radial bone defects were generated in 32 healthy adult rabbits. The defects were implanted with bone cement. The induced membranes and periosteum were removed after 2, 4, 6, and 8 weeks. Thereafter, hematoxylin-eosin staining (HE) and an enzyme-linked immunosorbent assay (ELISA) were performed to detect vascular endothelial growth factor (VEGF), angiotensin II (ANG-II), bone morphogenetic protein 2 (BMP2), fibroblast growth factor 2 (FGF2), and prostaglandin E2 (PGE2). Proteins isolated from total cell lysates were cultured with mesenchymal stem cells to test the cell proliferation and alkaline phosphatase activity using epimysium as a control.

**Results:**

The induced membrane and periosteum exhibited similar structures and growth factor levels after 4 and 6 weeks. The highest concentration of BMP-2 and VEGF in the induced membranes occurred in week 6, and FGF-2 and ANG-II concentrations peaked in week 4. The thickness and vascular density of induced membranes gradually decreased with time.

**Conclusion:**

Induced membrane matured between the 4th and the 6th week and secreted growth factors to promote osteogenesis. The matured induced membrane and periosteum had similar structures and abilities to promote the osteogenesis of mesenchymal stem cells. However, the induced membrane was thicker than the periosteum.

## Introduction

Previously, large bone defects especially those with severe soft tissue injury could only be treated by amputation. Advancements in technology have made limb salvage surgery more common [1]. However, bone reconstruction remains a major problem. Traditional methods for correcting bone defects include the autologous cancellous bone graft technique [2], the vascular bone graft technique [3], and bone transport [4]. Masquelet reported that long bone defects could be repaired by the use of an induced membrane in combination with an autologous cancellous bone graft, a technique known as induced membrane [5–8]. This technique involves the implantation of polymethyl methacrylate (PMMA) to induce the formation of membranes after one-stage radical debridement and reconstruction of bone defects using an autologous cancellous bone graft in a span of 4 to 8 weeks. Repairs to infections, radiation, and more complex bone defects up to 25 cm [8–11] were achieved using this technique. Bone cement implantation eliminated dead space to prevent infection and induced the formation of a membrane that exhibited a biological outcome. The membrane is primarily composed of type I collagen and fibroblasts, and the inner surface is rich in blood vessels [9, 12]. The membrane can secrete bone morphogenetic protein 2 (BMP-2), vascular endothelial growth factor (VEGF), core binding factor α1, interleukin 6, collagenase 1, and other growth factors to stimulate bone defect reconstruction [5, 7, 13, 14].

The morphological and molecular structural changes of the induced membrane over time suggested that the optimum time for grafting was from the 6th to the 8th week [5], but some studies demonstrated that osteogenic characteristics could persist from 4 weeks to 19 months [15, 16]. Other studies demonstrated that induced membranes might contain mesenchymal stem cells [7, 17], with some researchers having isolated and cultured mesenchymal stem cells from early polyethylene-induced membranes [18].

These findings revealed that prostaglandins (PGE2) might prevent bone resorption via the inhibition of osteoclast precursor differentiation [9, 13]. FGF-2 improves tissue angiogenesis and promotes osteogenesis [19, 20], while angiotensin 2 (ANG2) and VEGF both promote angiogenesis [21–23].

Periosteum is a rich vascular tissue because it contains numerous growth factors that promote osteogenesis and the healing of long bone fractures [24–26]. Two distinct levels are observed in the periosteum slice: the outer layer (fibrous tissue layer) and the inner layer (new layer) [27]. The epimysium surrounds the skeletal muscle with fibrillar connective tissue that consists of fibers and numerous other cell types. Isolated cells from the epimysium differentiate into chondrocytes in vitro [28].

This study aimed at ascertaining whether an induced membrane contained high concentrations of FGF-2, ANG2, VEGF, PGE2, BMP-2, and whether the levels of these growth factors changed over time. The structure and function of the induced membrane was compared to the periosteum and epimysium. The isolation of mesenchymal stem cells from the induced membrane was also attempted.

## Materials and methods

### Rabbit bone defect-induced membrane model construction

A total of 32 New Zealand rabbits, aged 3 to 4 months and with an average weight of 2.75 kg, were obtained from the Institute of Animals at the Third Military Medical University (Chongqing, China). Laboratory Animal Welfare and Ethics Committee Of the Third Military Medical University approved all of the animal protocols (SCXK-PLA-20120011). Fur was removed from the skin, and rabbits were restrained on an operating table and injected with 3 % sodium pentobarbital (1 ml/kg via ear vein; Merck KgaA, Germany). A 15-mm bilateral radial bone defect was produced using a saw. The periosteum in the defects was carefully removed and implanted with a 5 mm diameter, 15 mm long PMMA spacer (Smith nephew, Switzerland). Bone cement was used to wrap the ends of the bone defect, and the incision was sutured. Penicillin (20,000 IU/kg) was administered intramuscularly at 0, 24, and 48 h after surgery.

### Specimens

Eight rabbits were euthanized at week 2, 4, 6, and 8 with large doses of sodium pentobarbital, and 16 induced membrane specimens were obtained. Periosteum was harvested from the normal diaphyseal bone bilaterally, 2 cm away from the bone defect, and the epimysium was harvested from the biceps. All the operations were conducted under sterile conditions. Four samples from each group were stored in formalin for hematoxylin-eosin (HE) staining, and six samples were stored in liquid nitrogen for testing using an enzyme-linked immunosorbent assay (ELISA). Four samples were washed with PBS buffer for protein extraction and frozen at −80 °C. Two induced membrane samples were retained for cell isolation and culturing.

### HE staining

Membrane samples were stored in 10 % neutral formalin for 24 h, and 7 μm sections were generated for HE staining. Sections were visualized using a microscopy imaging system (Olympus, Japan) and IPP (Image Pro Plus) to measure membrane thicknesses.

### Protein extracts and ELISA

The membranes were weighed, added with protease inhibitors (Kangwei, Beijing, China) PBS at the ratio of 1:9 then homogenized, and the solution was centrifuged for 5 min at 12,000 rpm, 4 °C. The supernatants were used for ELISA and cell proliferation assay. Six induced membranes, periosteum, and epimysium samples were included in every assay. Each sample was tested for the concentration of BMP-2, VEGF, angiotensin II (ANG-II), FGF-2, and PGE2 using an anti-rabbit monoclonal antibody ELISA kit (Uscnk, Wuhan, China).

### Stem cell culture for proliferation

Cell viability was assessed by MTT assay. The mouse mesenchymal stem cell line C3H10T1/2 was donated by Professor Dong Shiwu and seeded in 96-well culture plates (1 × 104 cells/plate). Cells were then incubated with fresh medium containing 20 μl of protein extracts (the method of protein isolation from total cell lysates was described above) for 1, 3, 6, and 9 days. MTT was added into each well in a final concentration of 5 mg/ml for 4 h, dissolved in dimethyl-sulfoxide (DMSO) and measured with a rapid colorimetric assay at 570 nm of absorbed light.

### Osteogenic activity detection

C3H10T1/2 cells were seeded in 96-well culture plates (1 × 10^4^ cells/plate) and cultured in 0.2 ml of a DMEM/F12k medium (HyClone, USA) supplemented with 10 % fetal calf serum (FCS, Gibco, USA) and 4 μl of protein extracts that were obtained after 2, 4, 6, and 8 weeks. Medium without fetal bovine serum was used as the control. Alkaline phosphatase (ALP) activity was determined after 1, 3, 6, and 9 days using an ALP ELISA kit (Jiancheng, Nanjing, China). Enzyme activity is expressed as absorbance per microgram of protein per minute (μg/min/μgprot).

### Cell isolation and identification

The induced membranes were obtained under sterile conditions. Isolated membranes were finely chopped, washed three times in 0.01 mmol sterile PBS (pH 7.4), and suspended in a DMEM/F12k medium containing 10 % fetal bovine serum (HyClone, USA). The cultures were incubated in a 5 % CO_2_, 37 °C environment, and the culture medium was changed every 2 days using trypsin (HyClone, USA) digestion. The cultures were transferred to a new culture flask after 2 weeks. A total of 20,000 cells in the logarithmic growth phase were suspended in 0.1 ml of PBS-FCS and incubated with 1 μl of the FITC-conjugated mouse anti-rabbit monoclonal antibody CD 90 (Abcam, UK). The mouse anti-rabbit monoclonal antibodies CD 44 (ABD, UK) and CD 45 (ABD, UK) were visualized using 1 μl of a donkey anti-mouse FITC fluorescence secondary antibody (Kangwei, Beijing, China). The cells were washed twice with PBS for flow cytometry analysis. A total of 1 × 10^4^ cells/plate in the logarithmic growth phase were cultivated in osteogenic liquid which contained 1 × 10^−7^/l dexamethasone, 10 mol/l β-glycerol phosphate, and 50 mg/l of vitamin C for 14 days to measure ALP activity. A medium without induced fluid was used as a control.

Statistical Package for Social Sciences (SPSS) 19.0 software was used for the analyses. Statistical comparisons between two groups were performed using Student’s *t* test. Multiple groups were compared using a one-way analysis of variance (ANOVA). Data with an abnormal distribution was analyzed using the Kruskal-Wallis H test. A probability value of *P* < 0.05 was considered significant. Charts were generated using Graphpad Prism 5.

## Results

### Induced membranes gradually became thinner, and blood vessels were reduced over time

**Fig. 1:**
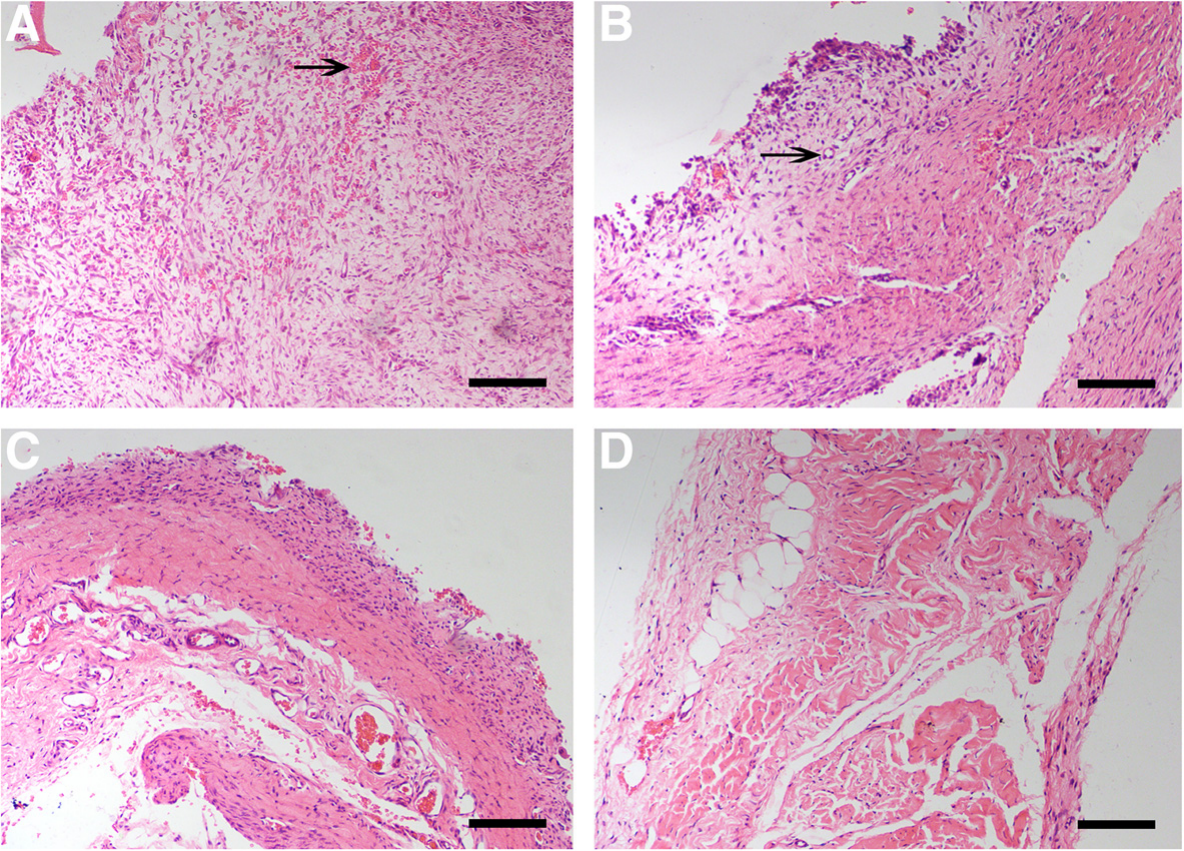
Induced membrane, periosteum, and epimysium sections with hematoxylin-eosin staining (×100). **a** A large number of capillaries and inflammatory cell infiltration at 2 weeks membrane. **b** Small blood vessels formed and vessel number decreased at 6 weeks. **c** The periosteum, inner layer of a large number of vessels and the outer layer of fibrous tissue. **d** The epimysium, no obvious hierarchy; contains blood vessels with fat vacuoles. *Black arrows* indicate blood vessels (Bar = 200 μm)

Induced membranes and periosteum contained two distinct layers: the inner layer, which is rich in blood vessels, and the outer layer, which is comprised of fibroblasts and collagen. However, the epimysium is just a single layer. At any time point assessed, the induced membranes were thicker than the periosteum and epimysium. The thickness of induced membranes decreased over time. Induced membranes were the thickest at 2 weeks (1360 ± 21 μm), and the membranes exhibited the most abundant vascularity with an inflammatory reaction. Vascularity was reduced and matured at 4 (1045 ± 26 μm) and 6 weeks (1008 ± 32 μm), and the periosteum was (890 ± 28 um) at week 8 (Fig. 1, Table 1). We observed edema, a large number of capillaries and inflammatory cell infiltration in the membrane, at 2 weeks.

**Table 1:** The thickness of the membranes (um)

	2 weeks	4 weeks	6 weeks	8 weeks
Induced membrane	1360 ± 21	1045 ± 26	1015 ± 19	1008 ± 32
Periosteum	887 ± 22	903 ± 17	899 ± 31	890 ± 28
Epimysium	912 ± 11	931 ± 20	895 ± 34	908 ± 18

### The levels of growth factors in induced membranes were similar to the periosteum at 4 and 6 weeks

**Fig. 2:**
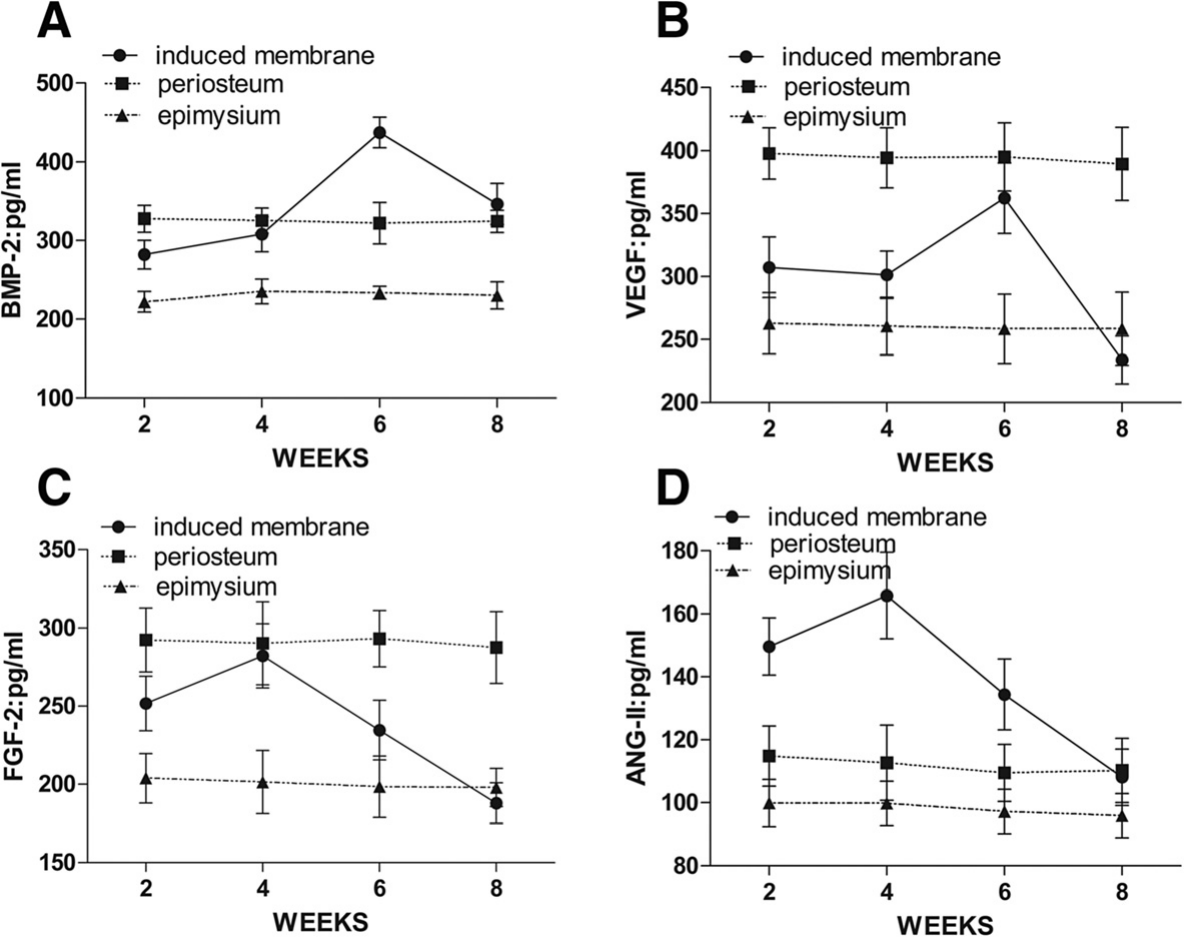
ELISA of growth factors in induced membranes, periosteum, and epimysium. **a** BMP-2 in induced membranes peaked at 4 weeks, higher than the periosteum and epimysium (*P* < 0.05). **b** VEGF in induced membranes peaked at 6 weeks and almost equaled the periosteum (*P* > 0.05). **c** FGF-2 in induced membranes peaked at 4 weeks (*P* > 0.05). **d** ANG-II in induced membranes peaked at 4 weeks (*P* < 0.05)

BMP-2, VEGF, ANG-II, and FGF-2 levels were lower in the epimysium at all time points (Fig. 2). BMP-2 (437.71 ± 19.14 pg/ml) and VEGF (362.35 ± 27.98 pg/ml) peaked in induced membranes after 6 weeks and then began to decline. The concentration of BMP-2 in induced membranes was significantly higher than the periosteum (322.25 ± 26.32 pg/ml) at 6 weeks (*P* < 0.05), but it was similar at all other time points. The VEGF concentration in induced membranes was similar to the periosteum at week 6 but was lower (395.15 ± 27.07 pg/ml) at the remaining points in time (Fig. 2a, b). FGF-2 (282.23 ± 20.53 pg/ml) and ANG-II (165.83 ± 13.67 pg/ml) peaked in the induced membranes after 4 weeks then began to decline. FGF-2 concentrations in the periosteum were always high, and the levels were similar to induced membranes after 4 weeks. ANG-II levels in induced membranes were higher than in the periosteum after 2, 4, and 6 weeks (Fig. 2c, d). However, no significant changes in ANG-II levels between the epimysium and periosteum were observed (*P* > 0.05). PGE2 levels in the induced membranes did not change significantly over time, and there were no significant differences between induced membranes, periosteum, and epimysium (*P* > 0.05).

### Induced membranes and periosteum exhibit similar properties in promoting stem cell proliferation and osteogenic differentiation during the maturation period

**Fig. 3:**
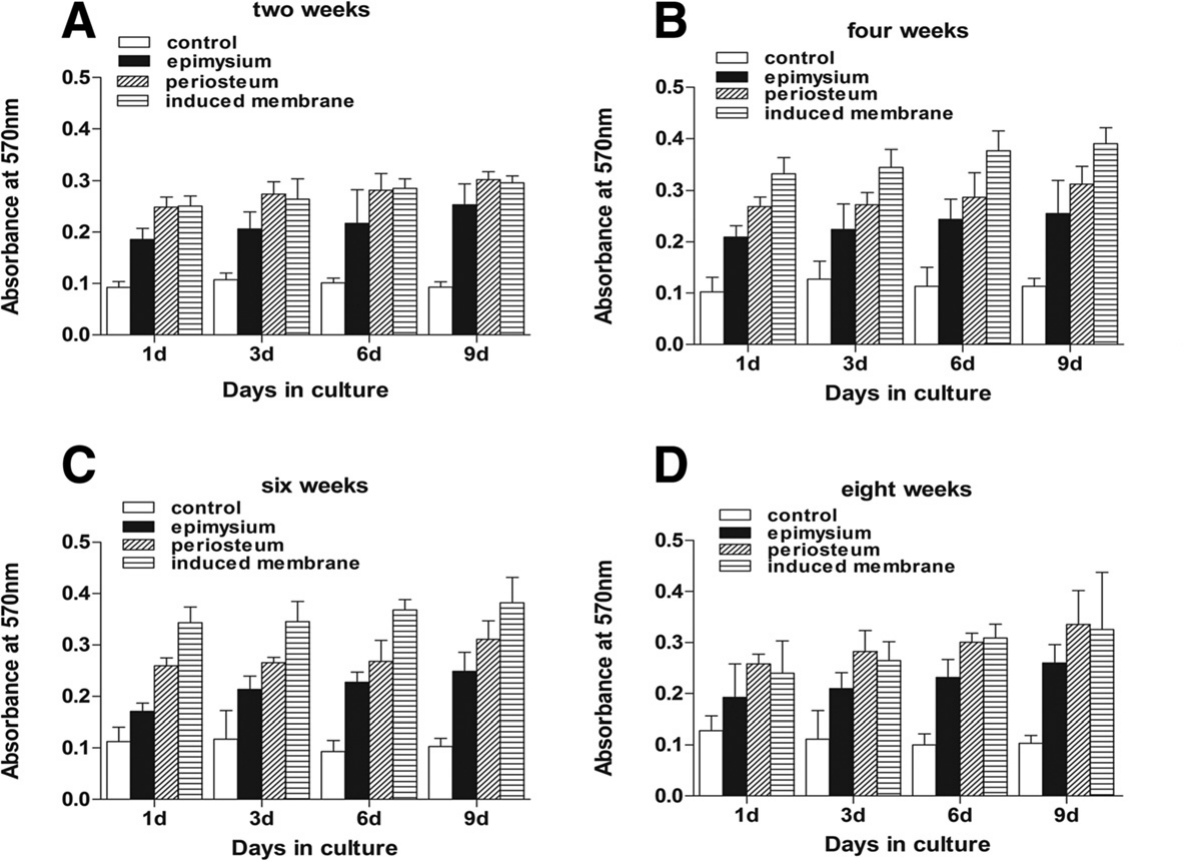
Proteins cultured with mouse mesenchymal stem cells to examine cell proliferation. **a**, **d** At weeks 2 and 8, the OD values for the induced membrane samples were almost equal to the periosteum (*P* > 0.05). **b**, **c** The OD values of the induced membrane samples were higher than the periosteum (*P* < 0.05)

**Fig. 4:**
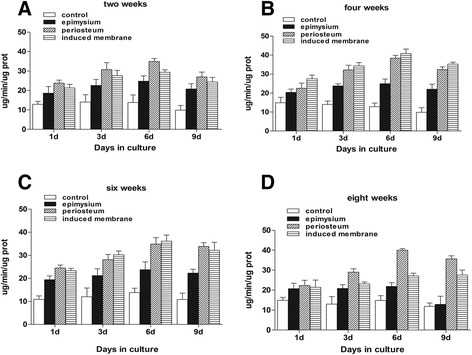
Alkaline phosphatase activity. **a**, **d** At weeks 2 and 8, the ALP activity in the periosteum group was higher than in the induced membrane group (*P* < 0.05). **b**, **c** Induced membrane and periosteum groups were almost equal (*P* > 0.05) and both were higher than the epimysium (*P* < 0.05)

**Fig. 5:**
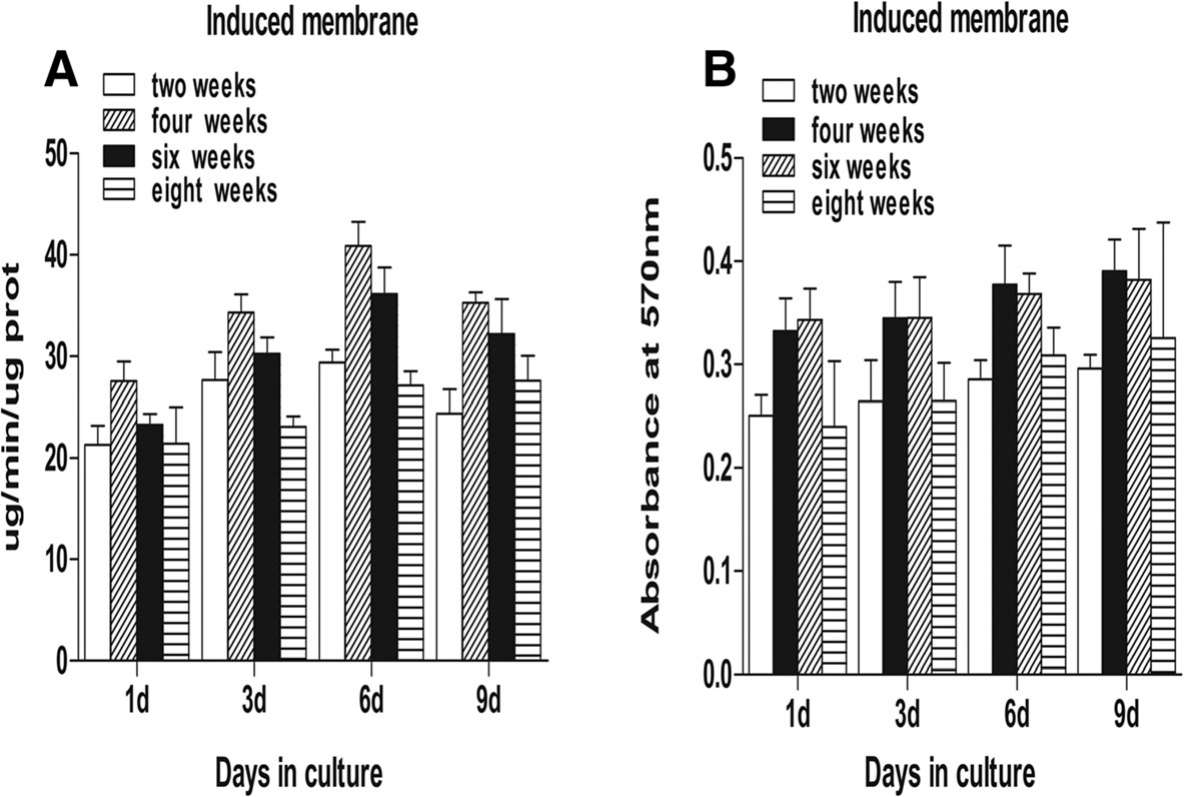
Comparison of induced membranes in the promotion of stem cell proliferation and osteogenic differentiation. **a** ALP activity was highest at week 4, lowest at weeks 2 and 8, and moderate at week 6 (*P* < 0.05). **b** OD values at weeks 4 and 6 were higher than at weeks 2 and 8 (*P* < 0.05)

This study demonstrated that mesenchymal stem cells in a culture medium containing total cell lysates isolated from the epimysium proliferated at a lower level as measured by absorbance at 570 nm. The ability of an induced membrane to promote mesenchymal stem cells was similar to the periosteum at weeks 2 and 8 (*P* > 0.05). The ability of induced membranes to stimulate cell proliferation was greater than the periosteum at weeks 4 and 6 (*P* < 0.05) (Fig. 3). Mesenchymal stem cells in osteoblasts were evaluated using alkaline phosphatase activity. The ALP activity in induced membrane was lower than in the periosteum at weeks 2 and 8 (*P* < 0.05). The ALP activity in induced membranes at weeks 4 and 6 was similar to the periosteum (Fig. 4). However, the stem cell proliferation generated by induced membranes was the strongest, and the ALP activity in the induced membranes was the highest after 4 weeks compared to other time points (Fig. 5).

### Isolated CD44^+^ cells exhibit osteogenesis

Induced membrane specimens were isolated after 2, 4, 6, and 8 weeks, and cultured adherent cells were fusiform or polygonal which contains one nucleus. Cells proliferated quickly and did not require stringent conditions. Flow cytometry detected positive rates of CD44 at 98.5 %, CD90 at 15 %, and CD45 at 1.35 %. ALP activity was significantly higher in induced membranes than the control group after 2 weeks in culture (*P* < 0.05).

## Discussion

The treatment of large bone defects remains challenging for surgeons. Induced membrane technology is an innovative method that improves the reconstruction of segmental bone defects. This technique is successful in the repair of large bone defects [5, 10, 29–31]. Bone cement implantation eliminates dead space and prevents fibrous tissue invasion at the recipient site. Implantation also induces the formation of the surrounding membrane, which is part of the histological characteristics and molecular components that were studied previously [7]. The present study compared the structure and effects of induced membranes, periosteum, and epimysium on osteogenesis and observed the structural and functional changes of the membrane over time.

HE staining revealed that mature induced membrane and periosteum contained two layers: an inner layer rich in blood vessels and an outer layer of fibrous components. These findings are consistent with previous studies [32], but the induced membranes in our study were much thicker at all time points. Early studies suggested that induced membranes are a fibrous tissue with a rich blood supply and a structure that is maintained for a long time [16, 33]. However, our study demonstrated severe inflammation and many tiny blood vessels after 2 weeks, then the membrane thickness and vascularity gradually decreased over time to form a periosteum-like tissue. Therefore, we conclude that the induced membranes are a parallel to periosteal tissues that degenerate over time.

BMP-2 is an initial signaling molecule in osteogenic differentiation in mesenchymal stem cells. BMP-2 enriches bone progenitor cells and promotes progenitor cell osteogenic differentiation [34]. VEGF promotes angiogenesis at injury sites and participates in the mobilization of mesenchymal stem cells in bone marrow. BMP-2 and VEGF boost the osteogenic differentiation of mesenchymal stem cells [35, 36]. We detected high concentrations of BMP-2 and VEGF in induced membranes and periosteum. Previous studies reported peak concentrations of BMP-2 and VEGF in induced membranes after 4 weeks [7, 14], but our peak concentrations occurred during the 6th week. We also detected two new growth factors, FGF-2 and ANG-II, which promote angiogenesis or osteogenesis [19, 20, 23]. FGF-2 and ANG-II reached peak concentrations in the 4th week in rabbits, but it might be different for humans. Therefore, we considered weeks 4 to 6 as the best grafting period, which conforms to the stem cell proliferation and osteogenic differentiation results. Previous studies reported a high concentration of prostaglandins in the prosthesis of joint replacement, which might play an important role in the prevention of absorbed bones [9, 13]. However, our results demonstrated that PGE2 content was not very high, and its effect in induced membranes might be exaggerated.

Our study demonstrated that protein extracts from induced membranes included growth factors that promoted the proliferation and differentiation of stem cells [7]. Our study also indicated that the ability of induced membranes to promote osteogenic differentiation of stem cells was similar to the periosteum at 4 to 6 weeks, but the content of BMP-2 in induced membranes was significantly higher at 6 weeks than the periosteum. These results indicate that there may be other significant factors involved in the promotion of bone formation. The ability of induced membranes to promote stem cell proliferation was stronger at the 4th to 6th week, which implies that the most appropriate time for two-stage grafting is weeks 4 to 6 according to our rabbit models. However, infection must also be controlled during the reconstruction of osteomyelitis and infected bone defects. Therefore, grafting time might be delayed for these patients.

Many studies have demonstrated that induced membranes might contain mesenchymal stem cells [14, 18, 37]. Henrich D et al. [38] found that the membranes induced at the femur defect (at 2 weeks) and in periosteum contain mesenchymal stem cells. These multi-potent cells differentiate into osteogenic and chondrogenic cells in animals and humans [32, 39]. Mesenchymal stem cells have not previously been isolated from induced membranes. Our study successfully isolated adherent cells from induced membranes for the first time, but we failed to demonstrate that these cells were mesenchymal stem cells. However, flow cytometry detected a positivity rate for the cell surface marker CD44 of 98.5 %, and these cells differentiated into osteogenic cells under an inducing agent. Therefore, we concluded that induced membranes likely promoted the repair and reconstruction of bone defects at defect sites, but this hypothesis requires further analysis.

The study was limited because it was performed on small animals, and the results might be different in human trials. Furthermore, this study primarily examined the structure of three types of membranes, but there was no exploration of the mechanisms of growth factor action. This study also failed to identify the cells that were isolated from the induced membranes.

## Conclusions

Induced membranes matured at the 4th to 6th week and secretion of growth factors to promote osteogenesis; the matured induced membrane and periosteum have similar structures and abilities to promote the osteogenesis of mesenchymal stem cells. We therefore concluded that the best time for grafting was at week 4 to 6.

## References

[1] Bosse MJ, MacKenzie EJ, Kellam JF, Burgess AR, Webb LX, Swiontkowski MF (2002). An analysis of outcomes of reconstruction or amputation after leg-threatening injuries. N Engl J Med.

[2] Keating JF, Simpson AH, Robinson CM (2005). The management of fractures with bone loss. J Bone Joint Surg Br.

[3] Pelissier P, Casoli V, Demiri E, Martin D, Baudet J (2000). Soleus-fibula free transfer in lower limb reconstruction. Plast Reconstr Surg.

[4] Cattaneo R, Catagni M, Johnson EE (1992). The treatment of infected nonunions and segmental defects of the tibia by the methods of Ilizarov. Clin Orthop Relat Res.

[5] Masquelet AC, Fitoussi F, Begue T, Muller GP (2000). Reconstruction of the long bones by the induced membrane and spongy autograft. Ann Chir Plast Esthet.

[6] Masquelet AC, Obert L (2010). Induced membrane technique for bone defects in the hand and wrist. Chir Main.

[7] Pelissier PH, Masquelet AC, Bareille R, Pelissier SM, Amedee J (2004). Induced membranes secrete growth factors including vascular and osteoinductive factors and could stimulate bone regeneration. J Orthop Res.

[8] Masquelet AC (2003). Muscle reconstruction in reconstructive surgery: soft tissue repair and long bone reconstruction. Langenbecks Arch Surg.

[9] Taylor BC, French BG, Fowler TT, Russell J, Poka A (2012). Induced membrane technique for reconstruction to manage bone loss. J Am Acad Orthop Surg.

[10] Accadbled F, Mazeau P, Chotel F, Cottalorda J, Sales de Gauzy J, Kohler R (2013). Induced-membrane femur reconstruction after resection of bone malignancies: three cases of massive graft resorption in children. Orthop Traumatol Surg Res.

[11] Giannoudis PV, Faour O, Goff T, Kanakaris N, Dimitriou R (2011). Masquelet technique for the treatment of bone defects: tips-tricks and future directions. Injury.

[12] Woon CY, Chong KW, Wong MK (2010). Induced membranes—a staged technique of bone-grafting for segmental bone loss: a report of two cases and a literature review. J Bone Joint Surg Am.

[13] Klaue K, Knothe U, Anton C, Pfluger DH, Stoddart M, Masquelet AC (2009). Bone regeneration in long-bone defects: tissue compartmentalisation? In vivo study on bone defects in sheep. Injury.

[14] Aho OM, Lehenkari P, Ristiniemi J, Lehtonen S, Risteli J, Leskela HV (2013). The mechanism of action of induced membranes in bone repair. J Bone Joint Surg Am.

[15] Apard T, Bigorre N, Cronier P, Duteille F, Bizot P, Massin P (2010). Two-stage reconstruction of post-traumatic segmental tibia bone loss with nailing. Orthop Traumatol Surg Res.

[16] Masquelet AC, Begue T (2010). The concept of induced membrane for reconstruction of long bone defects. Orthop Clin North Am.

[17] Ren L, Kang Y, Browne C, Bishop J, Yang Y (2014). Fabrication, vascularization and osteogenic properties of a novel synthetic biomimetic induced membrane for the treatment of large bone defects. Bone.

[18] Patel J, Gudehithlu KP, Dunea G, Arruda JA, Singh AK (2010). Foreign body-induced granulation tissue is a source of adult stem cells. Transl Res.

[19] Nomi M, Miyake H, Sugita Y, Fujisawa M, Soker S (2006). Role of growth factors and endothelial cells in therapeutic angiogenesis and tissue engineering. Curr Stem Cell Res Ther.

[20] Kim MS, Bhang SH, Yang HS, Rim NG, Jun I, Kim SI (2010). Development of functional fibrous matrices for the controlled release of basic fibroblast growth factor to improve therapeutic angiogenesis. Tissue Eng Part A.

[21] Etoh T, Inoue H, Tanaka S, Barnard GF, Kitano S, Mori M (2001). Angiopoietin-2 is related to tumor angiogenesis in gastric carcinoma: possible in vivo regulation via induction of proteases. Cancer Res.

[22] Fiedler U, Scharpfenecker M, Koidl S, Hegen A, Grunow V, Schmidt JM (2004). The Tie-2 ligand angiopoietin-2 is stored in and rapidly released upon stimulation from endothelial cell Weibel-Palade bodies. Blood.

[23] Parikh SM, Mammoto T, Schultz A, Yuan HT, Christiani D, Karumanchi SA (2006). Excess circulating angiopoietin-2 may contribute to pulmonary vascular leak in sepsis in humans. PLoS Med.

[24] Arnsdorf EJ, Jones LM, Carter DR, Jacobs CR (2009). The periosteum as a cellular source for functional tissue engineering. Tissue Eng Part A.

[25] Yu YY, Lieu S, Lu C, Colnot C (2010). Bone morphogenetic protein 2 stimulates endochondral ossification by regulating periosteal cell fate during bone repair. Bone.

[26] De Bari C, Dell’Accio F, Vanlauwe J, Eyckmans J, Khan IM, Archer CW (2006). Mesenchymal multipotency of adult human periosteal cells demonstrated by single-cell lineage analysis. Arthritis Rheum.

[27] Fan W, Crawford R, Xiao Y (2008). Structural and cellular differences between metaphyseal and diaphyseal periosteum in different aged rats. Bone.

[28] Li G, Zheng B, Meszaros LB, Vella JB, Usas A, Matsumoto T (2011). Identification and characterization of chondrogenic progenitor cells in the fascia of postnatal skeletal muscle. J Mol Cell Biol.

[29] Pannier S, Pejin Z, Dana C, Masquelet AC, Glorion C (2013). Induced membrane technique for the treatment of congenital pseudarthrosis of the tibia: preliminary results of five cases. J Child Orthop.

[30] Karger C, Kishi T, Schneider L, Fitoussi F, Masquelet AC (2012). Treatment of posttraumatic bone defects by the induced membrane technique. Orthop Traumatol Surg Res.

[31] Stafford PR, Norris BL (2010). Reamer-irrigator-aspirator bone graft and bi Masquelet technique for segmental bone defect nonunions: a review of 25 cases. Injury.

[32] Chang H, Knothe Tate ML (2012). Concise review: the periosteum: tapping into a reservoir of clinically useful progenitor cells. Stem Cells Transl Med.

[33] Viateau V, Guillemin G, Calando Y, Logeart D, Oudina K, Sedel L (2006). Induction of a barrier membrane to facilitate reconstruction of massive segmental diaphyseal bone defects: an ovine model. Vet Surg.

[34] Weidemann A, Johnson RS (2008). Biology of HIF-1alpha. Cell Death Differ.

[35] Chen L, Tredget EE, Wu PY, Wu Y (2008). Paracrine factors of mesenchymal stem cells recruit macrophages and endothelial lineage cells and enhance wound healing. PLoS One.

[36] Lin Z, Wang JS, Lin L, Zhang J, Liu Y, Shuai M (2014). Effects of BMP2 and VEGF165 on the osteogenic differentiation of rat bone marrow-derived mesenchymal stem cells. Exp Ther Med.

[37] Cuthbert RJ, Churchman SM, Tan HB, McGonagle D, Jones E, Giannoudis PV (2013). Induced periosteum a complex cellular scaffold for the treatment of large bone defects. Bone.

[38] Henrich D, Seebach C, Nau C, Basan S, Relja B, Wilhelm K, et al. Establishment and characterization of the Masquelet induced membrane technique in a rat femur critical-sized defect model. J Tissue Eng Regen Med. 2013. 10.1002/term.1826 24668794

[39] Gruber HE, Riley FE, Hoelscher GL, Bayoumi EM, Ingram JA, Ramp WK (2012). Osteogenic and chondrogenic potential of biomembrane cells from the PMMA-segmental defect rat model. J Orthop Res.

